# Courtship vocalizations in male ducks: spectral composition and resonance of the syringeal bulla

**DOI:** 10.1242/jeb.250117

**Published:** 2025-11-03

**Authors:** Darcy Mishkind, Paul R. Kaneelil, Michael Lester, L. Mahadevan, Clifford J. Tabin, Franz Goller

**Affiliations:** ^1^ Department of Genetics, Harvard Medical School, Boston, MA 02115, USA; ^2^ John A. Paulson School of Engineering and Applied Sciences, Harvard University, Cambridge, MA 02138, USA; ^3^ School of Biological Sciences, University of Utah, Salt Lake City, UT 84112, USA; ^4^ Department of Organismic and Evolutionary Biology, Harvard University, Cambridge, MA 02138, USA; ^5^ Department of Physics, Harvard University, Cambridge, MA 02138, USA; ^6^ Institute for Integrative Cell Biology and Physiology, University of Münster, 48149 Münster, Germany

**Keywords:** Syrinx, Sexual dimorphism, Helmholtz resonator, Vocal tract

## Abstract

Ducks display a unique and dramatic sexual dimorphism in their vocal organ, the syrinx. Males have a left-sided bulla that is not present in females and that has been long hypothesized to play a role in courtship vocalizations, though this connection has never been tested. The large, hollow morphology of the bulla and its proximity to the sound-producing vocal folds introduce the possibility that it may work as a Helmholtz resonator, which makes it possible to predict the resonance frequencies enhanced by this structure. We found that during early ontogeny, the distribution of energy across the harmonic spectrum of contact calls is not different between males and females. We then used microcomputed tomography (µCT) scans of duck syringes to estimate resonance frequencies of the bullae and compared these with spectral features of their vocalizations. This comparison overall supports the idea that the bulla resonance may specifically enhance aspects of courtship vocalizations, especially in species that have a tonal courtship whistle. This was further supported when we tested the frequencies produced when air was blown through 3D printed bullae. We also saw potential influence of the bulla in non-courtship vocalizations, which could be explored further with a greater understanding of the input of other vocal tract features that influence vocalization. We observed that, in general, excepting the common eider, bulla size shows a weak positive correlation with male bird body mass. This study provides support for the long-held hypothesis that the adult male duck bulla influences resonance frequencies, in particular in courtship vocalizations.

## Teaser

**Summary:** Evidence that the bulla - a left-sided hollow outgrowth on the avian vocal organ, the syrinx - in male ducks serves to influence resonance frequencies in male courtship vocalization.

## INTRODUCTION

Birds generate sound with a unique, avian-specific vocal organ located at the tracheobronchial junction, called the syrinx. The syrinx consists of modified cartilage elements of the trachea and bronchi, which house sound-generating vibratory tissue (King, 1989; Goller and Larsen, 1997; Elemans et al., 2015). Depending on the specific location of these vocal folds (often also called membranes or labia) in the tracheal or bronchial parts of the syrinx, birds can have one, two or three sound sources (King, 1989; Goller, 2016; Garcia et al., 2017). It is likely that a syrinx with two bronchial sound generators represents the original syrinx morphology (Clarke et al., 2016; Longtine et al., 2024), a vocal structure found in many avian orders, including the basal galloanserines. The vocal folds are suspended in a framework of calcified cartilages, which are often modified from typical bronchial or tracheal elements. High levels of variation in these structures inspired scientists early on to use the syrinx as a taxonomic characteristic for classifying birds (Wunderlich, 1884; Fürbringer and Fürbringer, 1888; Gadow and Selenka, 1891). While modern taxonomic methods have largely replaced these early classification systems, morphological characteristics of the syringeal skeletal elements still inspire research on the evolution of this unique vocal organ.

The morphological variety of the syrinx and vibratory tissues contributes to the large range of avian vocalizations seen in nature (Gaunt, 1983; Riede and Goller, 2014; Goller, 2022). In many avian taxa, vocal behavior also differs between males and females. However, we know relatively little about the degree to which these differences are rooted in sexual dimorphism of the vocal apparatus itself. Although the syringeal structures of male zebra finches are larger than those of females (Düring et al., 2013), analysis of differences in volume, fiber type composition and contraction dynamics of syringeal musculature indicate that neuromuscular control predominantly accounts for different acoustic output (Wade and Buhlman, 2000; Elemans et al., 2008; Christensen et al., 2017).

The most striking sexual dimorphism in syringeal structure can be found in ducks. The syrinx of male ducks exhibits a bulb-like, air-filled outgrowth of the left syringeal skeleton, referred to as a bulla (Rüppell, 1933; Johnsgard, 1961; King, 1989; Frank et al., 2007). In mallards (*Anas platyrhynchos*), the bulla is present at hatching, but sexual dimorphism of the syringeal labia and membranes is only expressed later (>45 days post-hatching) (Gottlieb and Vandenbergh, 1968; Warner, 1971; Lockner and Youngren, 1976). While duckling calls do not appear sexually dimorphic, adult male and female ducks of many species have distinct vocal repertoires (Abraham, 1974; Lockner and Murrish, 1975). In particular, in many species, males produce distinct courtship vocalizations (Lorenz, 1953; Johnsgard, 1971). It is tempting therefore to associate the presence of a bulla with the generation of courtship vocalizations (Johnsgard, 1961, 1971). While several possible roles of the bulla in the production or modification of vocalizations in the respective vocal repertoires have been proposed (Johnsgard, 1971; Lockner and Youngren, 1976), they have, to our knowledge, not been thoroughly tested.

How could the presence of a bulla influence vocal production? Ducks possess two sound generators (membranes or labia), and the bulla opening is situated close to the left labial pair. In mallards, the labia and attached medial tympaniform membranes are also sexually dimorphic, being small in females and thick in males (Warner, 1971). It is likely that oscillations of the labia are the primary sound source, although there is some disagreement in the literature (discussed in King, 1989). Sound production in male mallards appears to be linked to the presence of the bulla, such that the right pair of labia is not engaged (Lockner and Youngren, 1976).

Irrespective of the precise oscillatory mechanism, the generated sound is modified by the resonance properties of the upper vocal tract structures. The tracheal tube, oropharyngeal–esophageal cavity (OEC), glottal opening, mouth, tongue and bill provide static and dynamically adjustable mechanisms for upper vocal tract filtering and thus contribute to the spectral properties of emitted sound (Riede et al., 2006; Ohms et al., 2010, 2012; Beckers, 2013; Kazemi et al., 2023). In male ducks, the bulla is also predicted to contribute specific resonance properties (Abs, 1969, 1970a,b; Warner, 1971; Abraham, 1974). Its morphology and proximity to the sound source suggest that it may act as a Helmholtz resonator. Its geometry should therefore allow predictions about its resonance properties and, thus, which spectral features of vocalizations are enhanced. The presence of a bulla only in males opens an intriguing opportunity to ask how this specific feature contributes to vocalization.

While its function is untested, an impressive diversity of bulla morphology has been described (Johnsgard, 1961). The wide array of sizes has produced a ‘natural experiment’: as the size varies, so too does the frequency a given bulla is predicted to amplify. Taking advantage of this, we compared the vocalizations of different species with the frequencies we would predict based on their bullae morphology, to test whether there is evidence that the bulla is used for male-specific sound production. We made use of a wide collection of duck species found in a museum collection to produce microcomputed tomography (µCT) scans of duck syringes. We hypothesized that the bulla acts as a resonance chamber. Air flows past the bulla opening during phonation, which suggests strongly that it may act as a Helmholtz resonator, akin to the classical model of airflow past the opening of a bottle. The resonance of the bulla could therefore enhance certain frequencies and act as a filter. Here, we tested this model. Application of an alternative model used in other systems, in which the upper airway is modeled as a series of resonating structures (Gardner et al., 2001; de Boer, 2008, 2009; Riede et al., 2008), requires more anatomical information on upper vocal tract components (lengths and cross-sectional areas of tracheal segments and oropharyngeal space) than could be obtained from the museum specimens. On this basis, we used the scans to model which frequencies are enhanced by the bulla if it acts as a Helmholtz resonator and asked how these compare to the recorded vocal repertoires of a given species. For four species, two with tonal courtship whistles and two with more complex courtship vocalizations, we 3D printed models of the syringes and tested how the frequencies produced by the bullae as air was blown through them matched predictions and recordings. Because of the strong sexual dimorphism displayed in this trait, we expected it to play a role in the spectral composition of duckling calls and male-specific adult vocalizations.

## MATERIALS AND METHODS

### Duckling vocalizations

All procedures were approved by the Institutional Animal Care and Use Committee of the University of Utah. Mallard ducklings were ordered from a commercial breeder and kept in the laboratory in a pen equipped with an infrared heating lamp in one corner. They were fed *ad libitum* with commercial food for ducklings and were provided with water at all times. Starting at the age of 4 days, individuals were isolated in a recording chamber whose walls were lined with acoustic foam, for audio recording of contact calls (sometimes also called isolation or distress calls) (Kear, 1968). Approximately 100–500 contact calls were recorded with an Audiotechnica AT3032 omnidirectional microphone at 44.1 kHz with Avisoft-RECORDER. After the recording sessions, body mass was determined to the nearest 0.1 g. Recording sessions for each individual were repeated every week. We recorded calls from 4 males and 4 females for 7 weeks. After this period, the vocal repertoire developed into more diverse vocalizations, and birds no longer readily produced the contact calls when isolated. Sex was determined either post-mortem or when sex was revealed after molting into breeding plumage in the following spring.

From each recording we selected 15 loud calls that were free of background noise. We analyzed the sounds using Praat software version 6.4.07 (http://www.praat.org/, 17 March 2024). For each call, we used the central segment to determine the mean fundamental frequency and the relative amplitudes of the fundamental frequency (FF), and the second (F2) and third (F3) harmonics from power spectra. To do this, we generated power spectra and extracted frequency and amplitude values.

### Comparative analysis

Syrinx specimens were sourced from the Harvard Museum of Comparative Zoology ornithology department (Table 1). Samples were all air dried at the time of collection and stored at ambient temperature. As all bullae were calcified, bulla size is not predicted to have changed in the drying process. Scanning with µCT was conducted with X-Tek HMXST225 X-ray imaging system (Nikon Metrology, Inc., Brighton, MI, USA). Acceleration voltage was 60–75 kV and filament current was 120–280 µA. Copper 0.1 filter was used for samples containing metal attachments: 342835 (red-breasted merganser), 340383 (smew), 340385 (tufted duck). A total of 3142 projection images were captured at 2000 pixels×2000 pixels (detector pixel size=−0.2 mm). Two frames were averaged with 1 s exposure time per frame.

**Table 1.** Microcomputed tomography (µCT)-scanned duck species, sample measurements and calculated resonance frequencies

Species	Common name	Catalog no.	Side	Bulla volume (mm^3^)	Opening diameter (mm)	Neck length (mm)	Resonance frequency (Hz)
*Aix sponsa*	Wood duck	MCZ:Orn:341876	Left	1240.86	5.75	0.86	3405.92
		MCZ:Orn:347504	Left	1266.41	4.96	0.8	3111.78
*Anas fulvigula*	Mottled duck	MCZ:Orn:342070	Left	3197.14	6.17	1.14	2157.74
*Anas platyrhynchos*	Mallard	MCZ:Orn:347156	Left	3549.71	9.7	0.54	2757.61
*Anas rubripes*	American black duck	MCZ:Orn:335525	Left	3508.36	8.01	1.54	2337.92
		MCZ:Orn:346547	Left	3678.73	9.44	1.96	2459.33
*Aythya americana*	Redhead	MCZ:Orn:346918	Left	2095.85	6.03	0.87	2691.36
*Aythya collaris*	Ring-necked duck	MCZ:Orn:341918	Left	2015.66	8.27	1.88	3079.34
*Aythya fuligula*	Tufted duck	MCZ:Orn:340385	Left	1997.09	8.22	0.86	3290.00
*Bucephala clangula*	Common goldeneye	MCZ:Orn:347155	Left	5233.93	4.08	3.8	1030.91
		MCZ:Orn:347155	Right	1308.1	6.28	2.05	3179.06
*Chloephaga rubidiceps*	Ruddy-headed goose	MCZ:Orn:343209	Left	6744.18	6.46	1.54	1479.81
*Dendrocygna autumnalis*	Black- bellied whistling duck	MCZ:Orn:340273	Left	1,055.14	2.96	1.07	2392.71
*Histrionicus histrionicus*	Harlequin duck	MCZ:Orn:366269	Left	3798.3	4.24	0.75	1647.77
		MCZ:Orn:336960	Left	4368.48	4.56	1.33	1506.11
*Lophodytes cucullatus*	Hooded merganser	MCZ:Orn:342699	Left	1242.79	5.24	0.84	3230.34
*Mergus albellus*	Smew	MCZ:Orn:340383	Left	2214.95	5.39	1.87	2242.77
*Mergus serrator*	Red-breasted merganser	MCZ:Orn:341891	Left	6969.61	6.98	1.17	1568.31
		MCZ:Orn:341891	Right	4734.47	5.93	1.18	1725.71
		MCZ:Orn:341905	Left	6640.54	7.52	1.36	1656.23
		MCZ:Orn:341905	Right	3956.18	6.31	1.26	1946.70
		MCZ:Orn:342835	Left	8431.59	8.9	1.37	1621.77
		MCZ:Orn:342835	Right	5280.2	8.9	1.84	1994.08
*Somateria mollissima*	Common eider	MCZ:Orn:336858	Left	1361.12	6.07	1.43	3197.39
		MCZ:Orn:337334	Left	1439.66	7.14	1.49	3417.18
		MCZ:Orn:342589	Left	1377.45	6.51	1.2	3377.43
		MCZ:Orn:342590	Left	1409.08	7.62	1.15	3676.69
		MCZ:Orn:342591	Left	1362.43	8.27	2.11	3695.10
*Tachyeres brachypterus*	Falkland steamer duck	MCZ:Orn:342206	Left	3296.15	8.22	1.37	2476.04
*Tadorna tadorna*	Common shelduck	MCZ:Orn:347540	Left	3694.86	8.34	1.36	2360.11
		MCZ:Orn:347540	Right	1772.15	8.32	1.22	3433.64

Data were reconstructed with Nikon CT Pro 3D software. Bulla volume, neck length and opening diameter were measured using Dragonfly Pro software, version 2022.2 (https://dragonfly.comet.tech/; Object Research Systems, Inc., Montréal, QC, Canada). Measurements were used to model the bulla as a Helmholtz resonator:
(1)

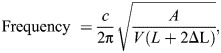
where frequency is in Hz, *c* is the speed of sound (343 m s^−1^), *A* is the neck opening area, *V* is the bulla volume and *L* is neck length. The calculation uses the following correction factor for short neck length relative to opening: 
(2)

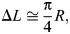
where *R* is the radius of the neck opening area.

A review of the literature indicated that several samples marked in the museum collections as female were, in fact, male (Bergmann and Düttmann, 2001; British Ornithologists’ Union, 1859; Miller et al., 2007; Wilson et al, 2013). Thus, we used a combination of museum annotations and literature to determine sample sex.

Recordings used for analysis were primarily collected from Cornell Lab of Ornithology's Macaulay Library. Any additional recordings required were gathered from xeno-canto.org as noted in Table 2. One to five separate recordings of each call type were combined to generate power spectra in Praat version 6.4.07 (http://www.praat.org/, 17 March 2024). Plots were smoothed with Cepstral smoothing set to 50 Hz.

**Table 2.** Sound files and their identifiers

Common name	Catalog no.	Call type	Recording no.	Sex	Recordist (for xeno-canto files)	Notes
American black duck	ML150360461	Call	1	F		
	ML433856921	Call	2	F		
	ML219819871	Call	3	F		
	ML315969151	Call	1	M		
	ML126638221	Call	4	M		
	ML328508111	Call	5	M		
	XC168801	Courtship	1	M	Paul Driver	Used for Fig. 5A
Black bellied whistling duck	ML89005	Call_1	1	NA		
	ML247999841	Call_2	2	NA		
	XC490269	Courtship	1	NA	Asher Warkentin	
	XC619234	Courtship	2	NA	Jayrson Araujo de Oliveira	
	XC690729	Courtship	3	NA	Jayrson Araujo de Oliveira	
	XC706125	Courtship	4	NA	Sue Riffe	
Common eider	ML235533	Call	1	NA		
	ML279182801	Call	2	NA		
	ML279182411	Call	3	NA		
	ML235533	Courtship	1	M		
	ML235533	Courtship	2	M		Used for Fig. 5B
	ML43067	Courtship	3	M		
Common shelduck	ML36267	Courtship	2	M		
	ML231075	Courtship	3	M		
	ML231085	Courtship	4	M		
	ML248054871	Call	1	F		
	ML352713301	Call	2	F		
	ML596491151	Call	4	F		
	ML359445041	Call	1	M		
	ML476574651	Call	2	M		
Falkland steamer duck	XC254304	Whistle	1	M	Andrew Spencer	
	XC254305	Whistle	2	M	Andrew Spencer	
	XC254304	Grunt	1	F	Andrew Spencer	
	XC254305	Grunt	2	F	Andrew Spencer	
Goldeneye	ML413534911	Call	1	NA		
	XC375849	Call	3	NA	Eetu Paljakka	
	XC504527	Call	4	NA	Lars Edenius	
	ML395683371	Courtship_A	2	M		
	ML408155701	Courtship_A	4	M		
	ML402828311	Courtship_A	5	M		
	ML551129461	Courtship_B	1	M		
	ML402827641	Courtship_B	3	M		
	XC715620	Courtship_B	6	M	Alan Dalton	
Harlequin	ML449381391	Courtship	1	M		
	ML610699420	Courtship	2	M		
Hooded merganser	ML414921191	Courtship	1	M		
	ML414920021	Courtship	2	M		Used for Fig. 5C
	ML414877771	Courtship	3	M		
	ML226731931	Courtship	5	M		
	ML567250191	Call	1	F		
	ML557997781	Call	3	F		
	XC317679	Flight	4	NA	Jim Berry	
	XC317868	Flight	5	NA	Jim Berry	
Mallard	ML228373231	Call	1	F		
	ML149645011	Call	2	F		
	ML296739821	Call	3	F		
	ML395598471	Flight	1	F		
	ML475387861	Flight	2	F		
	ML438183001	Flight	3	F		
	ML49536771	Call	1	M		
	ML439982151	Call	2	M		
	ML328909851	Call	4	M		
	ML404546811	Courtship	1	M		
	ML400751701	Courtship	2	M		
	ML446577071	Courtship	3	M		
Mottled duck	ML536156211	Call	2	F		
	XC451816	Call	3	F	Paul Marvin	
	ML186426991	Call	1	M		
	XC461530	Call	2	M	Paul Marvin	
Redhead	ML139672	Display	1	M		
	ML572034481	Display	2	M		
	ML573244651	Display	3	M		
	XC104592	Display	4	M	Andrew Spencer	
	ML92615411	Call	1	F		
	ML504647621	Call	2	F		
	XC78565	Call	3	F	Todd Wilson	
Red breasted merganser	ML140600801	Flight	1	M		
	XC655415	Song	1	M	Stein Ø. Nilsen	
	XC645027	Song	2	M	Stein Ø. Nilsen	
	XC134967	Song	3	M	Andrew Spencer	
	XC663253	Alarm_1	1	F	Sean Morris	
	XC488201	Alarm_2	2	F	Stanislas Wroza	
	XC449749	Call	1	F	Patrik Åberg	
Ring-necked duck	ML451513581	Courtship	1	M		
	ML68590	Courtship	2	M		
	ML53173	Annoyed	1	F		
	ML336115851	Annoyed	2	F		
	ML465097181	Annoyed	3	F		
	ML474624421	Annoyed	4	F		
	ML53515651	Quack	1	F		
	ML158773911	Quack	2	F		
	ML158773921	Quack	3	F		
	ML251252751	Quack	4	F		
	ML519031491	Quack	5	F		
	ML32239961	Call_1	1	M		
	ML100946041	Call_3	1	M		
	ML171263031	Call_3	2	M		
	ML162178571	Call_4	1	M		
	ML22485831	Call_5	1	M		
	ML430514431	Call_6	1	M		
Ruddy headed goose	ML24283901	Courtship	1	M		
	ML112864221	Call	1	M		
	XC89293	Call	2	M	Andrew Spencer	
	ML203976121	Diverse Call_2	1	M		
	ML242841161	Flight	1	M		
	XC44473	Call	1	F	Bernabe Lopez-Lanus	
Smew	XC618593	Courtship	1	M	Jarek Matusiak	
	XC610927	Courtship	2	M	Lars Edenius	
	XC565110	Courtship	3	M	Lars Edenius	
	XC127438	Alarm	1	M	Jarek Matusiak	
	XC618773	Alarm	1	F	Jarek Matusiak	
Tufted duck	XC800605	Courtship	1	M	Dominique Guillerme	
	XC730568	Courtship	2	M	Stanislas Wroza	Used for Fig. 7
	XC644316	Courtship	3	M	Lars Edenius	
	XC417298	Courtship	4	M	Joost van Bruggen	
	ML203913701	Call	1	M		
	ML65221741	Alarm	1	F		
	ML103332761	Alarm	2	F		
	ML557196541	Alarm	3	F		
	XC730568	Alarm	3	F	Stanislas Wroza	
	ML103332911	Flight	1	F		
Wood duck	ML108151031	Courtship_1	1	M		
	ML103919981	Courtship_2	2	M		
	ML110355	Courtship_2	3	M		
	ML220985281	Courtship_2	4	M		
	ML224564671	Call	1	M		
	ML545118281	Call	2	M		
	XC89717	Call	3	M	Mike Nelson	
	ML107967	Call	1	F		
	ML104878411	Call	2	F		
	ML326963961	Call	3	F		
	ML176235	Call	4	F		
	ML464904071	Call	5	F		

Sound files were used in Fig. 6 and Figs S1 and S2, as well as in other figures as noted. For catalog numbers, the prefix ‘ML’ denotes that it came from the Macaulay Library, ‘XC’ indicates it came from xeno-canto.org.

### 3D printing of syringes

We used the μCT scans to 3D print the duck bullae with a VisiJet M2R-CL (MJP) resin printer, achieving a resolution of approximately 35 μm. Before conducting air flow experiments with the printed bullae, we removed the trachea and sealed any artificial holes on the structure – caused by imperfections in the specimens – using tape and a layer of Play-Doh. To isolate the airflow pathway, we covered the secondary syringeal opening, leaving only the one nearest the bulla exposed.

Air flow experiments were done by passing air in the exhalation direction using a compressed air source or by the experimenter blowing across the bulla. This approach enabled us to probe a wide range of flow rates and confirm that the dominant frequency is independent of the details of the air flow and is only a function of the geometry of the bulla, as expected for a Helmholtz resonator. For each bulla, we conducted 5–7 trials, employing both air sources across repetitions. Audio was recorded using the built-in microphone of an iPhone 13 mini at a sampling rate of 48 kHz. A MATLAB code was used to perform Fourier analysis of the data and to calculate the dominant frequency ±1 s.d. Recordings were used to generate power spectra in Praat version 6.4.07 (http://www.praat.org/, 17 March 2024) (Boersma and Weenink, 2024). LPC smoothing was set to 30 peaks, 50 Hz pre-emphasis.

### Phylogeny generation and PGLS/PIC analysis

BirdTree.org was used to produce 100 trees of the species of interest with *Branta canadensis* (Canada goose) as an outgroup. Hacket All Species was used as the tree source (Hackett et al., 2008; Jetz et al., 2012). The Phytools package version 2.4.4 was used on R version 4.3.3 (Revell, 2024). Average and consensus trees were generated and found comparable. Regressions was conducted with phylogenetic generalized least squares (PGLS) and phylogenetically independent contrasts (PIC) models using both generated trees. All combinations yielded comparable results. The CRC handbook of avian body masses was used for male body mass data (Dunning, 2007). When more than one male mean body mass was listed for a species, the mean generated from the larger sample size was used. Graphing was done with GraphPad Prism 10 for macOS version 10.2.3 (347; 21 April 2024) unless otherwise noted.

## RESULTS

**Fig. 1.**
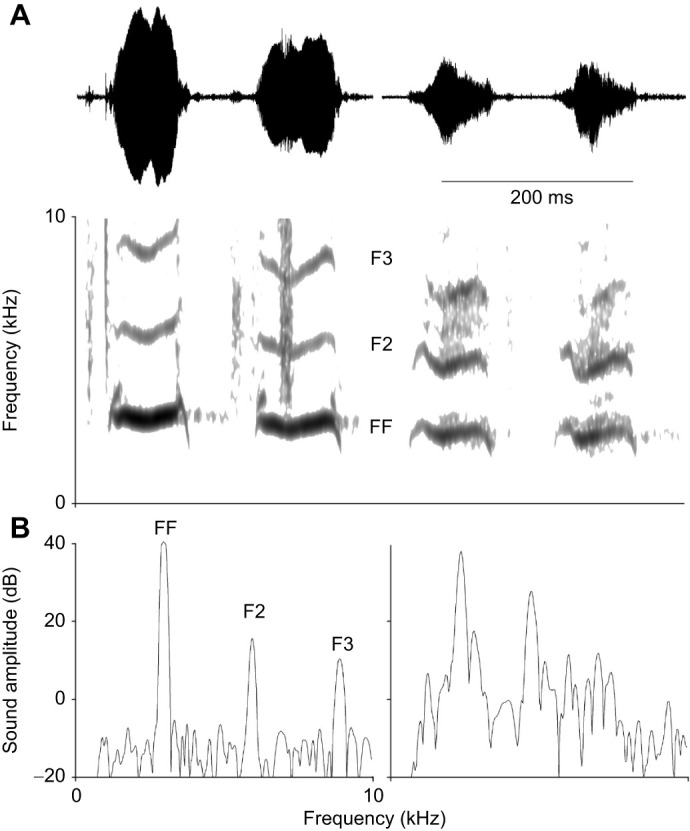
**Male duckling contact calls.** (A) Contact call examples (shown as an oscillogram and spectrogram) from a male duckling at 11 and 32 days of age, illustrating the decrease in fundamental frequency (FF) and the harmonic content (F2, second harmonic; F3, third harmonic). (B) Power spectra of the center region of the first calls at each age show the increased emphasis of the second harmonic at the older age (right).

**Fig. 2.**
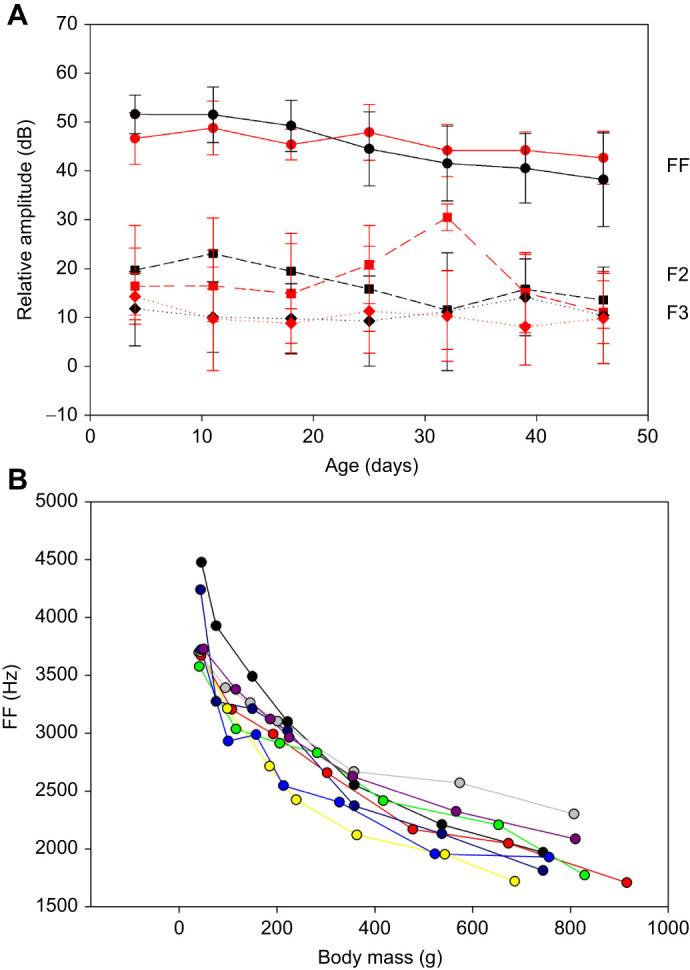
**Male and female duck contact calls show similar trends in amplitude and fundamental frequency over developmental time.** (A) Male (red) and female (black) contact calls show similar relative amplitudes (means±1 s.d.) of fundamental frequency (FF, circles and solid lines), and second (F2, squares and dashed lines) and third (F3, diamonds and dotted lines) harmonics over the age range. The exception is a more pronounced second harmonic at 32 days in male calls. Call amplitude declined slightly as ducklings tended to give softer contact calls with increasing age. (B) The FF (means) of contact calls declines as ducklings grow. Different colors are used for the data of different individuals.

We began by exploring the developmental progression of duckling contact calls in order to evaluate whether the presence of a bulla in males affects the spectral energy distribution. The frequency contour of contact calls was found to be hat-like with rising and declining frequency at the onset and offset (Fig. 1A). The fundamental frequencies had the highest relative amplitude (dB) and harmonics were typically not emphasized (Figs 1 and 2). Energy distributions across the measured time scale were very similar for males and females (Fig. 2A). The only exception was around age 32 days post-hatching, where the mean F2 amplitude in males was distinctly higher (approximately 20 dB) than that at all other time points and notably higher than that of females (Fig. 2A). At this time point, the FF of the four male ducklings ranged from 2.2 to 2.7 kHz (Fig. 2B), thus providing a range of the F2 from 4.4 to 5.4 kHz.

**Fig. 3.**
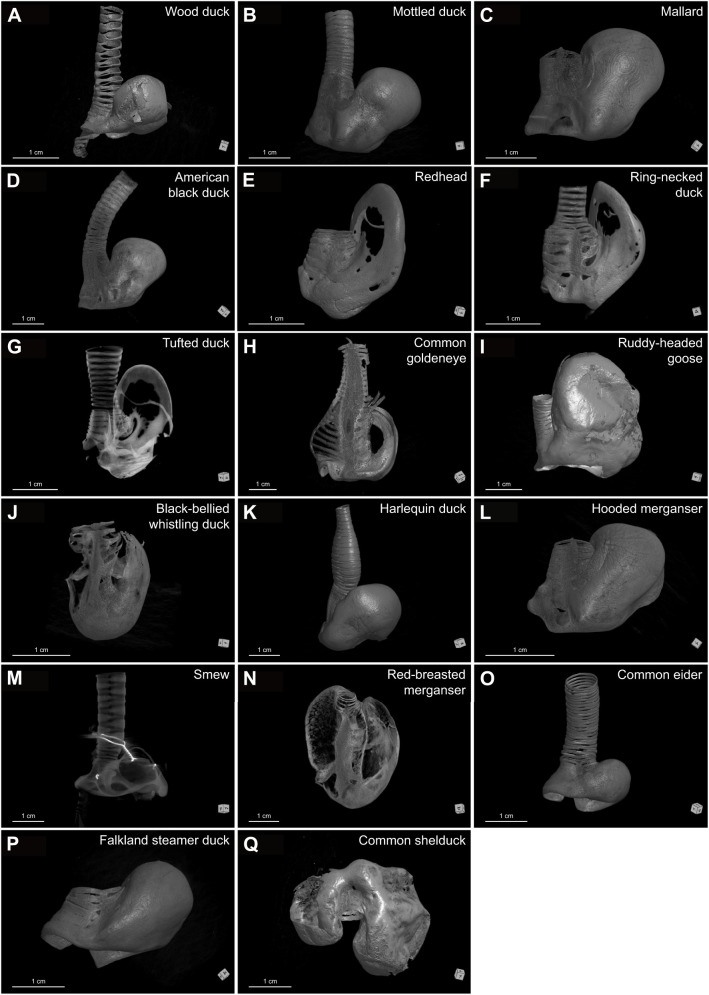
**Two-dimensional renderings of microcomputed tomography (µCT)-scanned syringes.** (A–Q) Ventral view of syringes from the following species (with sample indices): (A) wood duck (*Aix sponsa*), 341876; (B) mottled duck (*Anas fulvigula*), 342070; (C) mallard (*Anas platyrhynchos*), 347156; (D) American black duck (*Anas rubripes*), 335525; (E) redhead (*Aythya americana*), 346918; (F) ring-necked duck (*Aythya collaris*), 341918; (G) tufted duck (*Aythya fuligula*), 340385; (H) common goldeneye (*Bucephala clangula*), 347155; (I) ruddy-headed goose (*Chloephaga rubidiceps*), 343209; (J) black-bellied whistling duck (*Dendrocygna autumnalis*), 340273; (K) harlequin duck (*Histrionicus histrionicus*), 336960; (L) hooded merganser (*Lophodytes cucullatus*), 342699; (M) smew (*Mergus albellus*), 340383; (N) red-breasted merganser (*Mergus serrator*), 341905; (O) common eider (*Somateria mollissima*), 337334; (P) Falkland steamer duck (*Tachyeres brachypterus*), 342206; and (Q) common shelduck (*Tadorna tadorna*), 347540. The mallard image is reproduced for comparison (Mishkind et al., 2024 preprint). Scale bars: 1 cm.

We then turned to the potential function of the duck bulla. We took advantage of the Harvard Museum of Comparative Zoology ornithology collections to study 17 specimens of species of duck with intact syringes. We used µCT scans to establish the dimensions of the bullae (Fig. 3, Table 1; Fig. S3). Three of the species had a smaller right bulla in addition to the left bulla: the common goldeneye, red-breasted merganser and common shelduck (Table 1). These measurements were then used to model each bulla as a Helmholtz resonator and predict resonance frequencies (Table 1).

**Fig. 4.**
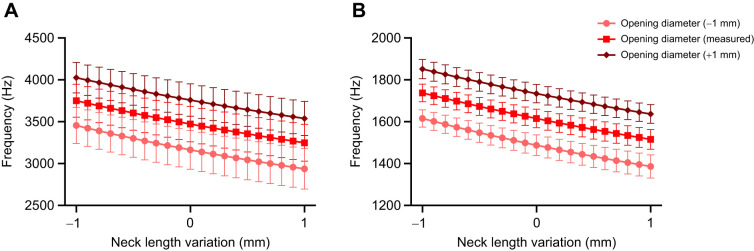
**Model of the influence of opening diameter and neck length measurements on predicted resonance frequencies.** Opening diameter was calculated for 1 mm smaller (pink circles) than measured (red squares) to 1 mm larger than measured (dark red diamonds). Neck length varied from 1 mm smaller than measured to 1 mm larger than measured. (A) Common eider (*n*=5). (B) Red-breasted merganser (left bulla, *n*=3). Error bars show ±1 s.d.

The bulla frequency predictions were based on three key measurements: the bulla volume, the opening diameter and the neck length of the opening. It is difficult to get precise estimates for the latter two variables, and the opening diameter may be subject to dynamic adjustment. For these reasons, we established how much our estimates for resonance frequency were altered by a 1 mm increase and decrease in these parameters. We used two example species. The change in opening diameter resulted in a maximum average change of 13% for red-breasted merganser and 14% for common eider. The change in neck length resulted in a maximum average change in length of 77% for red-breasted merganser and 71% for common eider. From this, we established that we could expect at maximum of approximately 500–1000 Hz range in resonance frequencies from a combined change in opening diameter and neck length. Opening diameter and neck length contributed approximately equally to the change in resonance frequency (Fig. 4).

**Fig. 5.**
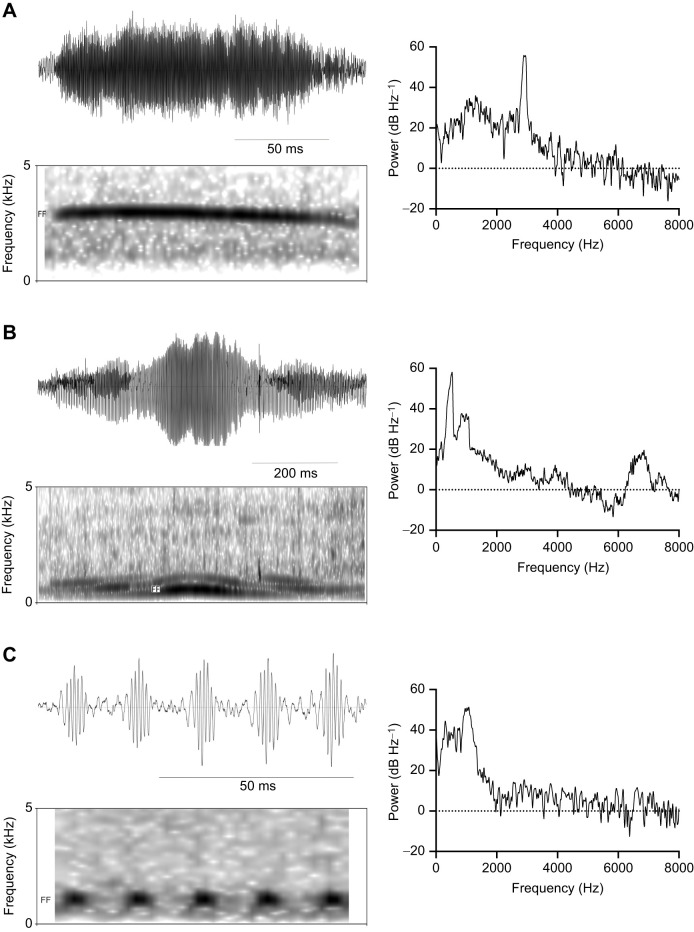
**Courtship call examples.** Left: oscillogram (top) and spectrogram (bottom); right: power spectra of the vocalizations shown on the left. (A) American black duck, which has a whistle-like vocalization. (B) Common eider, which has a low fundamental frequency and a complex call. (C) Hooded merganser, which has a low fundamental frequency and a simple call.

**Fig. 6.**
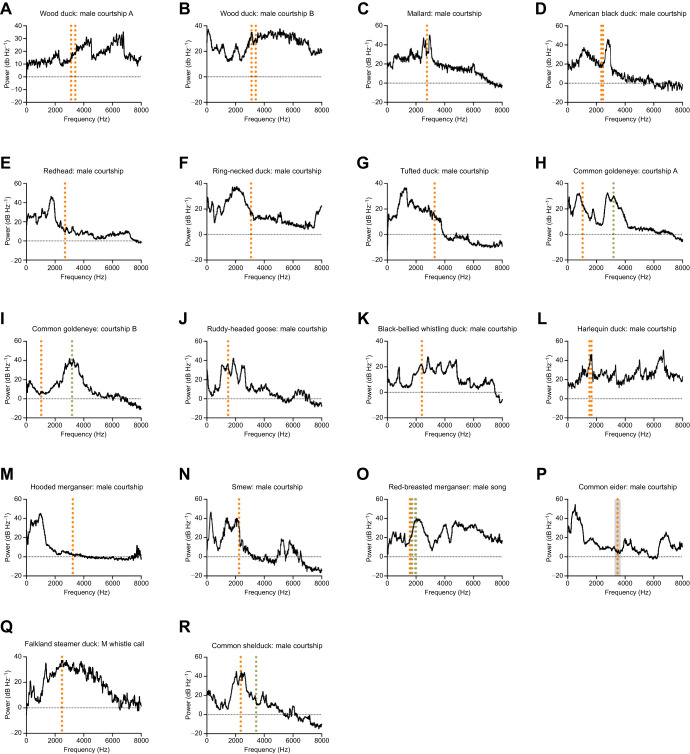
**Courtship vocalization power spectra compared with predicted resonance frequencies of the bulla.** Power spectra of 1–5 separate recordings were combined to produce power spectra for a given call type in the following species: (A) wood duck: male courtship A; (B) wood duck: male courtship B; (C) mallard: male courtship; (D) American black duck: male courtship; (E) redhead: male courtship; (F) ring-necked duck: male courtship; (G) tufted duck: male courtship; (H) common goldeneye: courtship A; (I) common goldeneye: courtship B; (J) ruddy-headed goose: male courtship; (K) black-bellied whistling duck: male courtship; (L) harlequin duck: male courtship; (M) common goldeneye: courtship B; (N) ruddy-headed goose: male courtship; (O) black-bellied whistling duck: male courtship; (P) harlequin duck: male courtship; (Q) Falkland steamer duck: M whistle call; and (R) common shelduck: male courtship. The vertical orange dotted line represents the predicted resonance frequency of the left bulla. The vertical gray dotted line represents the predicted resonance frequency of the right bulla. Additional dotted lines indicate predictions from additional samples. The mallard data are reproduced for comparison (Mishkind et al., 2024 preprint). Common eider, the only sample to have five replicates, is shown with gray shading representing ±1 s.d.

We then compared the spectral energy distributions of different call types across species and with the predicted resonance frequencies. Fig. 5 shows examples for call types from three species and the corresponding power spectra. The spectral energy distribution was then plotted and compared with the predicted bulla frequencies (Fig. 6; Figs S1 and S2). For 10 out of 16 species, the predicted resonance frequency of the bulla was within 500 Hz of a peak in the power spectrum, and in 13 out of 16 species it was within 1000 Hz (Fig. 6). The courtship calls of the mallard and closely related species are tonal, whistle-like vocalizations and in those, the bulla resonance was close to the fundamental frequency (Figs 5 and 6). The courtship calls of other species are characterized by low fundamental frequency and rich content of spectral energy in higher harmonics. In those, the predicted resonance frequencies of the bullae were close to one of the upper harmonics (Fig. 6; Figs S1 and S2). For the common goldeneye courtship call A, we saw an intriguing overlap of each predicted bulla frequency with a separate peak in the power spectrum, while in courtship call B, only the right bulla's resonance frequency overlapped with a peak (Fig. 6H,I). In the common shelduck, we were only able to observe the left bulla’s resonance frequency overlapping with a courtship vocalization peak, while we were unable to match the predicted frequency of the right bulla with any vocalization peaks (Fig. 6R).

**Fig. 7.**
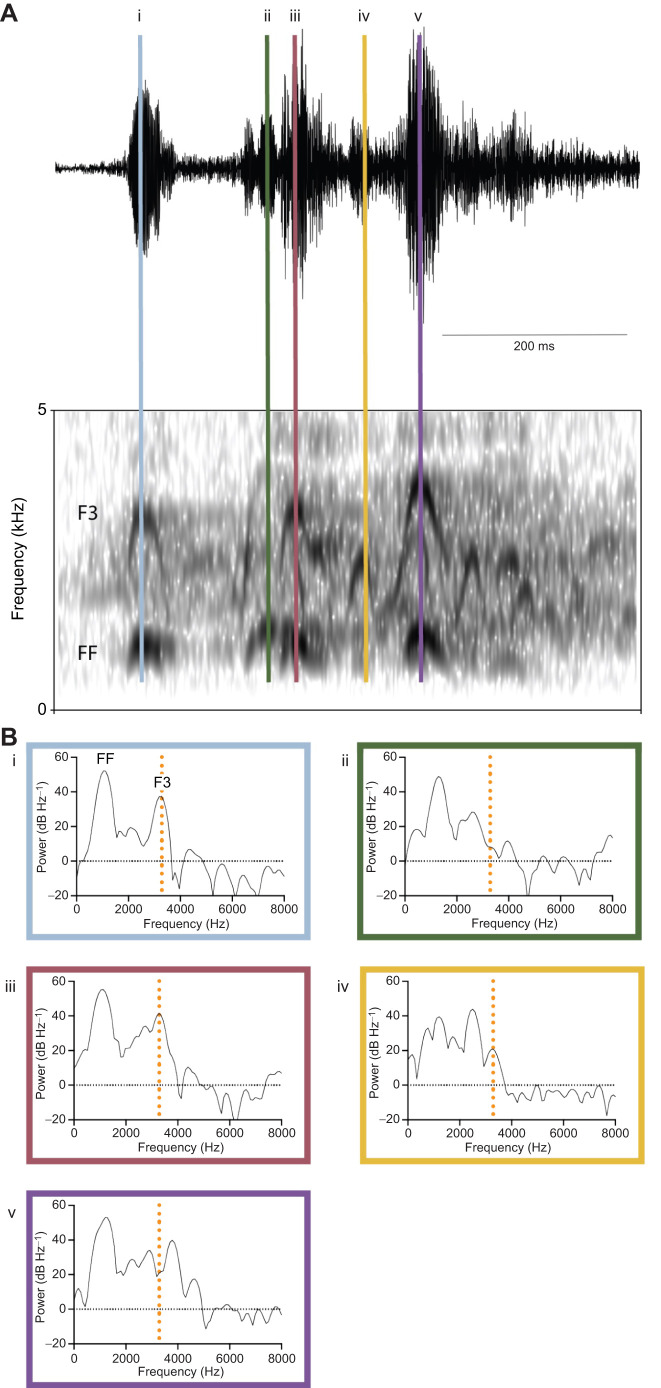
**Tufted duck courtship call is variable.** (A) Tufted duck courtship call shown as an oscillogram (top) and spectrogram (bottom). (B) Power spectra of single points in the vocalization (i–v). The vertical orange dotted line represents the predicted resonance frequency of the left bulla. FF, fundamental frequency; F3, third harmonic.

Finally, for three species (tufted duck, hooded merganser and common eider), we could not find a match between the spectral peaks of call types and the predicted resonance of the bulla. Tufted duck has a complex courtship call that when taken as a whole shows an initial peak followed by an irregular region at around 20 dB below the peak (Fig. 6G). However, the fundamental frequency is modulated throughout the call, which leads to an unclear distribution of energy for the power spectra across entire calls (Fig. 7). A different story emerged when we looked at spectra at single points in the vocalization: there were some where the fundamental frequency was emphasized, the second harmonic suppressed, and a more prominent third harmonic overlapped with our bulla prediction (Fig. 7).

In the case of the hooded merganser, the low frequency courtship calls are likely generated in pulse tone mode with pulse repetition frequencies of approximately 55–65 Hz and each pulse consisting of a series of damped oscillations with a frequency of 1200–1600 Hz. Surprisingly for pulse tone vocalizations (Jensen et al., 2007; Sitt et al., 2008), the upper harmonic content in these calls was very low. It is unclear whether the low upper harmonic content is in part due to the recording conditions (distance and noisy background) and/or reflects active suppression (Fig. 5C).

To further test the bulla as a Helmholtz resonator, we 3D printed the syringes of four species: mallard, American black duck, harlequin duck and wood duck. These four species had well-preserved syringes with the first two representing species with tonal courtship calls and the latter two having more complex courtship vocalizations (Fig. 6). When pressurized air was blown through the syringes or a human blew into the syringes, we observed similar frequencies to predicted and recorded peak frequencies for the two with tonal vocalizations, while no clear match to peaks was observed between the produced frequencies and the spectral energy distributions in the latter two species (Fig. S4). For Mallard and American black duck, we observed peak frequencies of 2850±402.5 Hz (sample 347256) and 2752±171.7 Hz (sample 346547), respectively, while measurements were not closely matched for harlequin duck (1944±903.1 Hz and 2263±47.0 Hz for samples 366269 and 336960) and wood duck (3585±710.1 Hz for sample 341876).

**Fig. 8.**
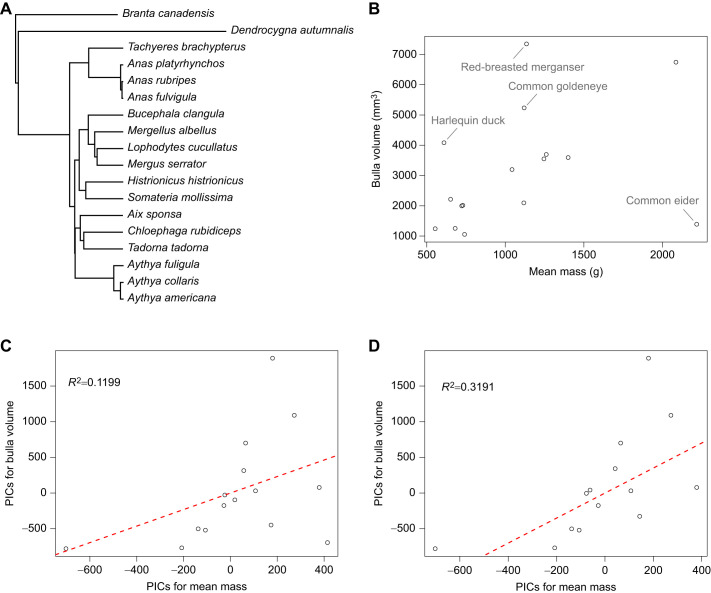
**There is a weak correlation between bulla volume and mean species body mass.** (A) Duck species phylogeny with Canada goose (*Branta canadiensis*) as an outgroup. (B) Bulla volume versus mean species body mass without any corrections for phylogeny. Species that fall outside the general trend are indicated. (C) Regression using phylogenetically independent contrasts (PIC) and average tree. The dashed red line represents the regression line (*P*=0.103; *R*^2^=0.1199; slope=1.1576). (D) Regression using PIC and average tree excluding common eider (*Somateria mollissima*). The dashed red line represents the regression line (*P*=0.0165; *R*^2^=0.3191; slope=1.7529).

We then asked whether bulla volume and male body mass showed a correlation. We hypothesized that outliers might represent species that had poor matches between our predicted bulla resonance frequencies and the recorded courtship calls. One striking outlier was the common eider, whose bulla is small relative to its body mass (Fig. 8). The common eider corresponds with our worst-matched estimate between courtship call and bulla resonance frequency (approximately 3 kHz difference; Fig. 6P). Overall, we observed a weak positive correlation between male mean body mass and bulla size. When the common eider was excluded, we saw a regression line with a slope of 1.7529 (*P*=0.0165; *R*^2^=0.3191) (Fig. 8). Two of the species that fell outside the general trend, the red-breasted merganser and common goldeneye, both showed large bulla volumes for birds of their mean body mass. Interestingly, both species have left and right bullae. However, the third species we studied that has both left and right bullae, the common shelduck, did fall within the general trend. The last species to fall somewhat outside the general trend, the harlequin duck, also has a moderately larger bulla volume than the others in relation to body mass.

Taken together, our results are consistent with the bulla functioning as a Helmholtz resonator, at least in most duck species. Moreover, our results support the idea that the bulla serves to influence resonance frequencies during male courtship vocalizations.

## DISCUSSION

Despite much speculation about the possible roles of the duck bulla, its functional relevance has not previously been explored. As a first step in that direction, we specifically questioned whether the bulla could function as a resonator to enhance courtship vocalizations. Predicted resonance frequencies for different species generally overlapped with peaks in the spectral energy of courtship vocalizations. However, we also saw matching of predicted and observed resonance peaks in other male vocalizations and in female calls. While the male resonance peaks in non-courtship vocalizations can of course also be attributed to the bulla, in females, other resonances such as those of the trachea and OEC must account for the specific filtering. This latter conclusion therefore suggests that specific spectral properties of vocalizations arise from combined, and possibly overlapping, action of the various components of the upper vocal tract.

The trachea constitutes a tube whose resonance can be estimated. Assuming that ducks do not actively change tracheal length very much (Fitch, 1999; Daley and Goller, 2004; Ohms et al., 2010), we can use the range of measurements to calculate resonance frequencies. In the mallard, tracheal length ranges between 14 and 18 cm (Klingler, 2016; Al-Ahmed and Sadoon, 2020), resulting in a resonance frequency of 476–613 Hz, if the trachea is seen as a tube with one open end. The fact that the fifth harmonic of the tracheal resonance frequency aligns with the predicted resonance frequency of the bulla may explain the overlap between our prediction and the peak frequency of the female mallard flight call. This highlights one difficulty of evaluating the bulla predictions. After sound is produced by the syrinx, many components of the vocal tract beyond the bulla, including the trachea (Abs, 1969, 1970a), glottal opening, OEC and beak opening, influence which frequencies are enhanced (Fletcher et al., 2006; Riede et al., 2006; Kazemi et al., 2023).

The comparison between male and female mallard ducklings provides a test of the extent to which the presence of a bulla in males alters the spectral distribution of energy in their vocalizations. We used contact (distress) calls, because these can be readily elicited by isolating the duckling and their context specificity allows direct comparison. Furthermore, they are uttered with high amplitude and are not contaminated by calls of other ducklings as would be the case in a social group. Surprisingly, there were no differences between calls from males and females with the exception of 1 month old birds. The 20 dB higher second harmonic of male calls could have arisen from a match with the resonance frequency of the bulla. Although we do not have measurements of the size of the bulla at this age, the 4–5 kHz resonance frequency suggests a volume of approximately 540 mm^3^ (for 5 kHz) to 840 mm^3^ (for 4 kHz) assuming neck length and opening diameter are half the adult measurements. Conversely, we can ask why at all other times the bulla did not produce a match with harmonics in the calls. Given the bulla is still growing during this period, it may be a result of the bulla resonance frequency not matching the call frequencies until this time. Measurements of the growing bulla over time would be necessary to assess this.

Age was not included in sample annotations of our museum specimens, though research in male king eiders and common eiders has shown that the size of the bulla does not vary between 1 year olds and adults (Miller et al., 2007). Furthermore, in the species for which the most individuals could be sampled, the common eider, we only found small individual variation (coefficient of variation=6.09%), which is consistent with published results in king and common eiders (Miller et al., 2007).

Our results constitute support for the hypothesis that the bulla of male ducks provides resonance properties that enhance aspects of their courtship vocalizations. We could only estimate the resonance frequencies because opening diameter and neck length of the opening could not be unambiguously determined. In addition, the skeletal elements in these museum specimens do not give insight into the degree to which dynamic adjustment of the opening plays a role. For example, the close proximity of the labia and other soft tissue (Warner, 1971) at the opening could lead to a changing opening diameter and different driving pressures could even facilitate its dynamic modulation. In addition, precise shape (King, 1989; Frank et al., 2007), soft tissue partial partitions within the bulla (Warner, 1971; Lockner and Youngren, 1976; King, 1989) and fenestrations covered by elastic soft tissue, as are found in the bullae of *Aythya* species (Warner, 1971; King, 1989), will also affect the resonance properties.

The comparative approach includes species with very different spectral properties of courtship calls, whistle-like calls, broad-band, harmonically rich calls and pulse tone calls. This broad range of spectral characteristics also leads to a potentially different role of bulla resonance across our duck species. Indeed, analysis of frequencies produced from 3D printed bullae support that its function may vary between species with whistles compared with those that produce more harmonically rich courtship calls. While tuning to the whistle frequency will enhance the total amplitude of the courtship call, in harmonically rich courtship calls, bulla resonances can contribute to enhancing specific frequency bands in the harmonic content. Finally, bulla resonances could also act as filters of specific frequencies, as may be the case in the low-frequency pulse tone calls. The wealth of acoustic characteristics of courtship calls in the Anatidae points toward an interesting evolutionary relationship between the marked sexual dimorphism in syringeal structure and vocal behavior. Although we do not know which acoustic properties of courtship vocalizations are ancestral within Anatidae, it is possible that harmonically rich calls represent original courtship vocalizations. Whistled courtship calls are prominent in the genus *Anas* and may therefore be derived, which is consistent with their phylogenetic position within Anatidae (Sun et al., 2017). Resonance properties of the bulla can contribute to the broad range of vocal properties. The variation of bulla volume relative to male body mass points toward interesting species in this context. However, it is also possible that the evolution of vocal features has outpaced changes to the morphological structure and therefore we are seeing evolution of syringeal morphology in progress.

Here, we tested whether the bulla might act as a Helmholtz resonator, which could contribute specific resonances for enhancing particular frequencies of vocalizations arising from labial oscillations. Our comparative approach yielded correlational evidence that largely supports this hypothesis. The proposed model and our findings therefore match previous applications of the Helmholtz resonator function (e.g. Suthers et al., 1988; Mindlin and Laje, 2005; Sitt et al., 2010; Uribarri et al., 2020). While such a function of the bulla is the most plausible, the present evidence cannot rule out some of the proposed alternatives.

Another model for determining filter properties of vocal tracts, taking into account their composition as a series of segments with different geometries (e.g. Gardner et al., 2001; De Boer, 2008, 2009), could not be tested here, because the upper vocal tract was not preserved in our specimens. However, theoretical considerations suggest that this alternative model may not describe the role of the bulla better than the model used here.

The main uncertainty stems from the fact that we have very little physiological data on the sound production mechanisms in ducks. For example, it is not clear which anatomical structures are the main sound generators. While some authors identify the labia, others postulate various membranes as the primary sound sources (Rüppell, 1933; Warner, 1971; King, 1989). An additional unsubstantiated claim is that the bulla itself may generate sound as air flows past or through it. As a potential whistle mechanism, Johnsgard proposed an aeolian whistle (Johnsgard, 1961, 1971). Aeolian whistles generate sound as vortices are shed when air flows around a cylinder (Chanaud, 1970), and it may therefore not be the most likely mechanism in light of the morphology of the male duck syrinx. Alternatively, sound could be generated with a whistle mechanism that resembles that of human whistling (Azola et al., 2018) or a flow-excited Helmholtz resonator, akin to an airstream flowing over the opening of a bottle. In this latter case, the resonance properties of the bulla, as calculated here, will predict the generated frequencies. While whistle mechanisms are clearly not the source of any vocalizations with complex harmonic spectra, one of them could give rise to the courtship whistles within the genus *Anas*. Future work will be needed to determine whether different production mechanisms give rise to different vocalizations within the call repertoires of ducks.

## Supplementary Material

10.1242/jexbio.250117_sup1 Supplementary information

Table S1. Dois of all μCT scans used in the study.
