# Supplementary material for: Courtship vocalizations in male ducks: spectral composition and resonance of the syringeal bulla
Source: J Exp Biol. 2025 Nov 3;228(21):jeb250117. doi: 10.1242/jeb.250117 (PMC12633744; doi:10.1242/jeb.250117)
Supplement: Supplementary information [file jexbio-228-250117-s1.pdf]

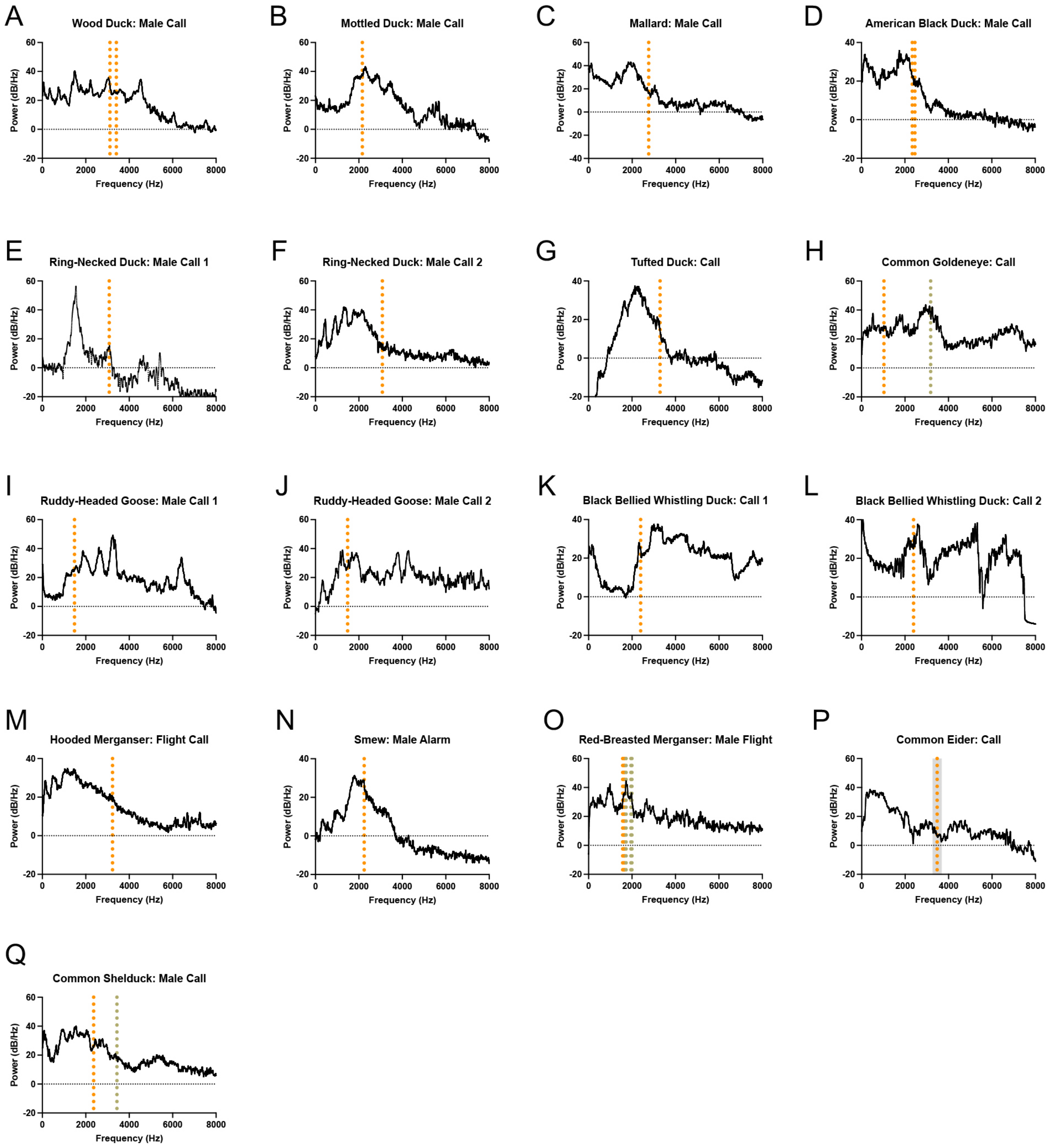

Fig. S1. Male non-courtship vocalization power spectra compared to predicted resonance frequencies of the bulla. Power spectra of one to five separate recordings were combined to produce power spectra for a given call type. Vertical orange dotted line represents the predicted resonance frequency of the left bulla. Vertical grey dotted line represents the predicted resonance frequency of the right bulla. Additional dotted lines indicate predictions from additional samples. Mallard is reproduced for comparison (Mishkind et al., 2024). Common eider, the only sample to have five replicates, is shown with grey shading representing ± 1s.d..

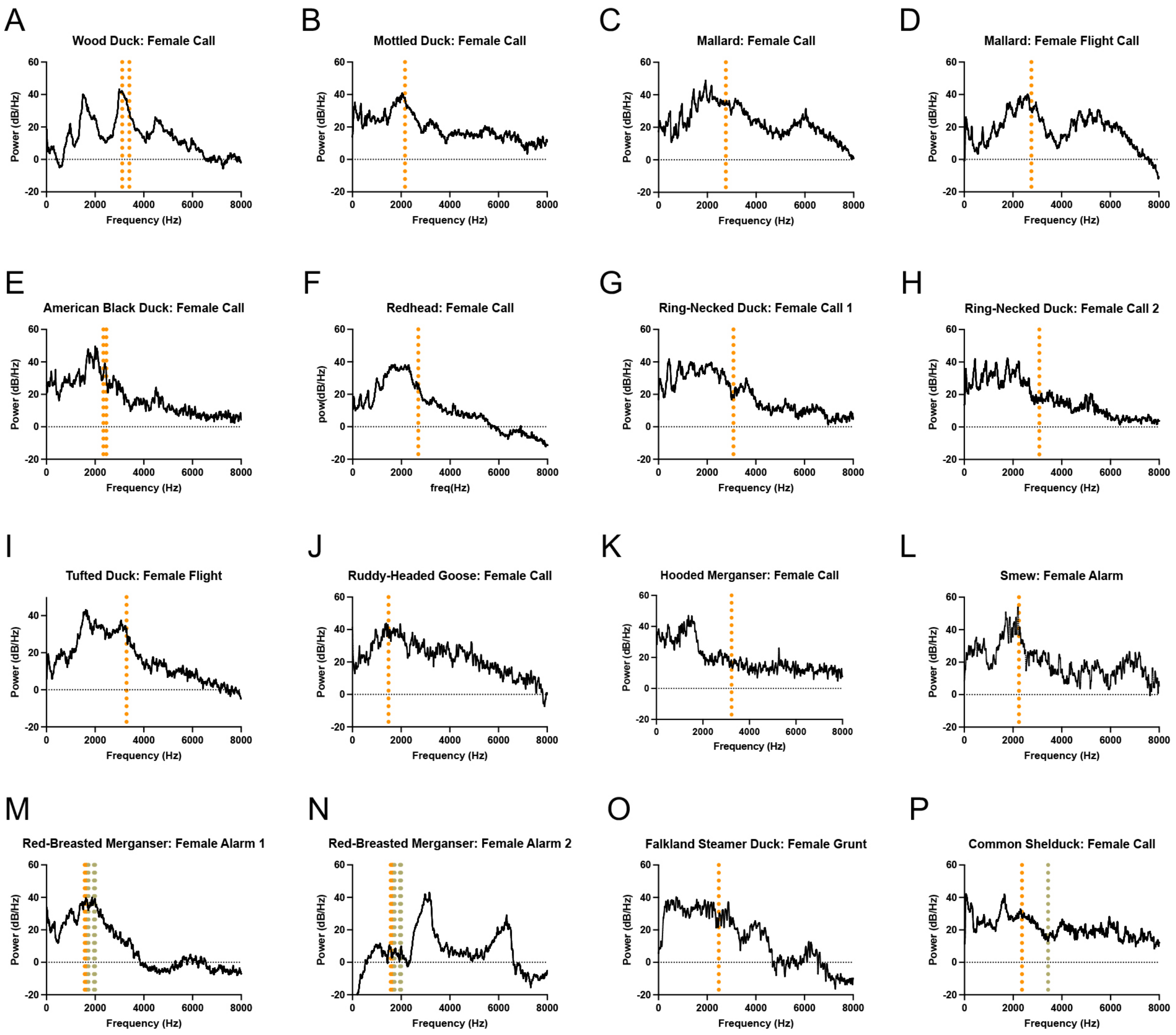

Fig. S2. Power spectra of female vocalizations compared to predicted resonance frequencies of the bulla. Power spectra of one to five separate recordings were combined to produce power spectra for a given call type. Vertical orange dotted line represents the predicted resonance frequency of the left bulla of conspecific males. Vertical grey dotted line represents the predicted resonance frequency of the right bulla. Additional dotted lines indicate predictions from additional samples. Bulla resonance in the Common Eider, for which five male bullae were sampled, is shown with grey shading representing Ñ1s.d..

Journal of Experimental Biology • Supplementary information

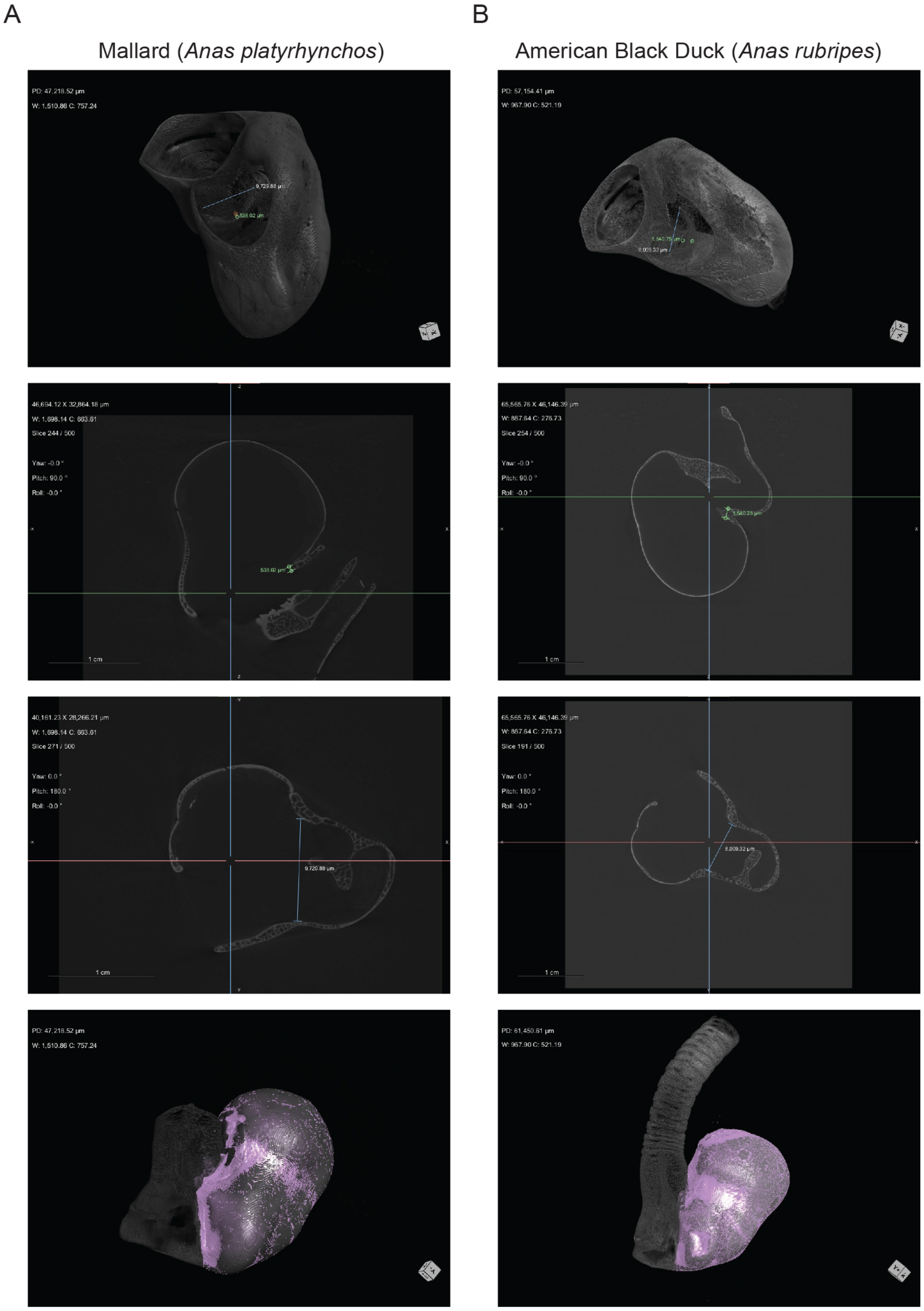

Fig. S3. Examples of neck length, opening diameter, and bulla measurements. (A) Mallard (sample 347156) and (B), American Black Duck (sample 335525) showing (top to bottom) an overview of measurements, cross-sectional view of neck length measurement, crosssectional view of opening diameter measurement, and purple highlight of volume selected to measure bulla volume.

Journal of Experimental Biology • Supplementary information

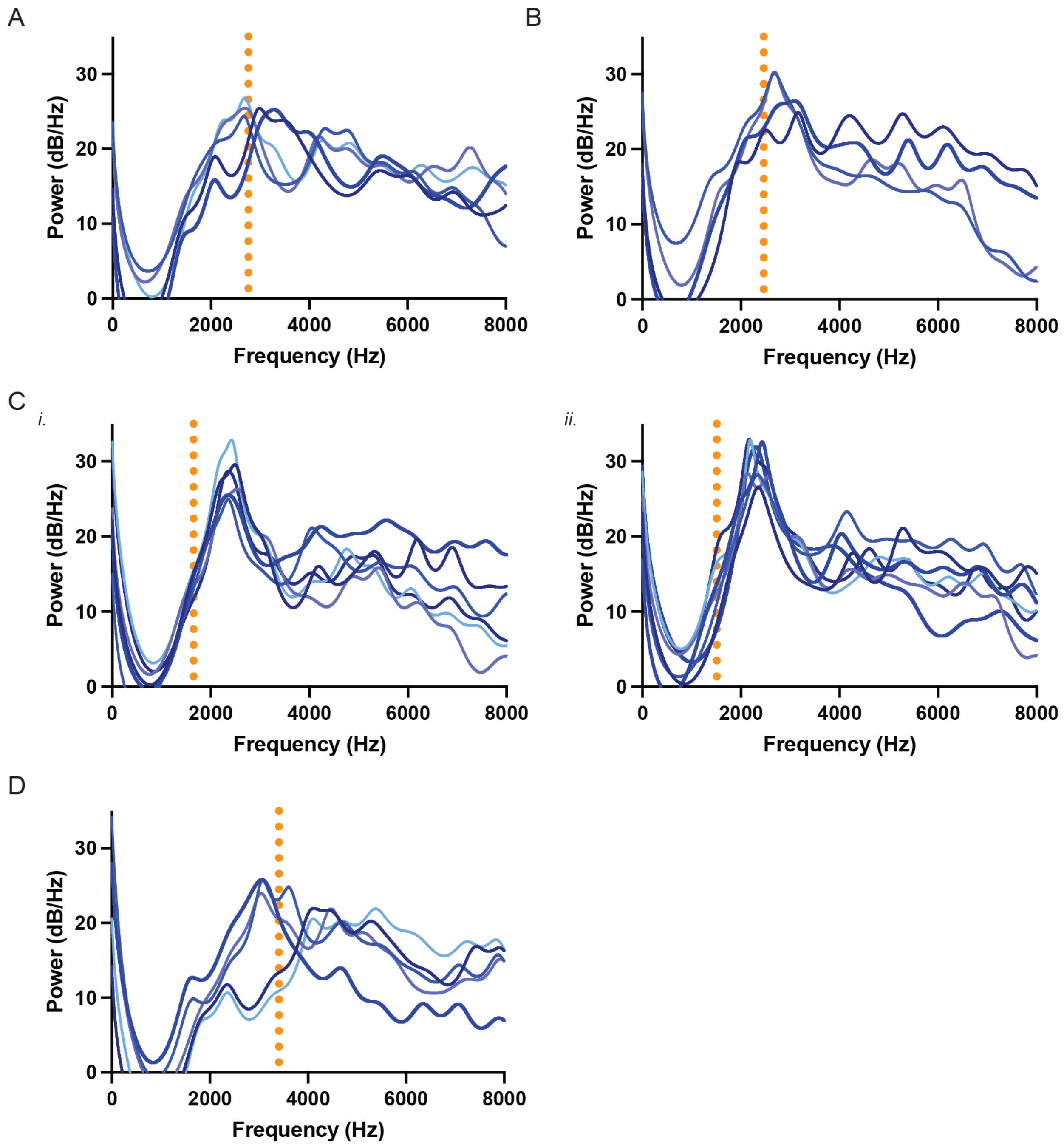

Fig. S4. Power spectra from 3D-printed syrinx flow experiments. (A) Mallard (sample 347156); (B) American Black Duck (sample 346547); (C) Harlequin duck i., sample 336269, ii., sample 336960; (D) Wood duck (sample 341876). Replicates are shown in varying shades of blue. Vertical orange dotted line represents the predicted resonance frequency of the sample's bulla.

Table S1. Dois of all μCT scans used in the study.

Available for download at
https://journals.biologists.com/jeb/article-lookup/doi/10.1242/jeb.250117#supplementary-data
